# Spontaneous neural activity alterations in medication-naïve primary blepharospasm: a resting-state functional magnetic resonance imaging study

**DOI:** 10.3389/fnsys.2025.1639915

**Published:** 2025-07-14

**Authors:** Hua-Liang Li, Shu Wang, Xin-Xin Yao, Si-Yu Gu, Jian-Bin Hu, Ping-Lei Pan

**Affiliations:** ^1^Department of Neurology, Affiliated Hospital 6 of Nantong University, Yancheng Third People’s Hospital, Yancheng, China; ^2^Department of Radiology, Affiliated Hospital 6 of Nantong University, Yancheng Third People’s Hospital, Yancheng, China; ^3^Department of Central Laboratory, Affiliated Hospital 6 of Nantong University, Yancheng Third People’s Hospital, Yancheng, China

**Keywords:** primary blepharospasm, amplitude of low frequency fluctuation, putamen, thalamus, premotor cortex, resting-state functional magnetic resonance imaging

## Abstract

**Background:**

Brain functional reorganization in primary blepharospasm (BSP) remains incompletely understood. This study aimed to add to the increasing knowledge by examining abnormalities in local spontaneous neural activity in this disorder.

**Methods:**

Resting-state functional magnetic resonance imaging data were acquired from 32 medication-naïve patients with BSP and 32 age- and sex-matched healthy controls in this study. The imaging data were analyzed using the amplitude of low frequency fluctuation (ALFF) to measure spontaneous neural activity. Partial correlation analyses between the altered ALFF values and clinical variables (illness duration and Jankovic Rating Scale score) in patients with BSP were further conducted.

**Results:**

Compared to healthy controls, medication-naïve patients with BSP exhibited significantly increased ALFF in the bilateral putamen and left premotor cortex and decreased ALFF in the bilateral thalamus (*p* < 0.05, threshold-free cluster enhancement with family-wise error correction for multiple comparisons). Furthermore, ALFF values in the left putamen in the patient group were positively correlated with illness duration (*r* = 0.53, *p* = 0.002).

**Conclusion:**

Our findings reveal aberrant spontaneous neural activity within key regions of the motor control network in medication-naïve BSP patients. These ALFF alterations, especially the progressive changes observed in the putamen, provide novel insights into BSP neuropathophysiology and highlight the value of studying untreated cohorts to understand the disorder’s intrinsic characteristics.

## Introduction

Primary blepharospasm (BSP) is one of the most common primary dystonic disorders, characterized by involuntary closure of the eyelids mainly due to the spasms of the orbicularis oculi muscles (Defazio et al., 2017). Beyond its characteristic motor manifestations, BSP is frequently associated with non-motor symptoms, including sensory, psychiatric, sleep, and cognitive disturbances (Defazio et al., 2017; Ferrazzano et al., 2019; Valls-Sole and Defazio, 2016). BSP is a progressive condition that can cause functional disability and reduce quality of life (Defazio et al., 2017; Ferrazzano et al., 2019; Valls-Sole and Defazio, 2016). Diagnosis is primarily clinical, as established biomarkers are lacking. Converging evidence from neurophysiological and neuroimaging studies implicates a disordered brain network, encompassing the basal ganglia, thalamus, cortex, and cerebellum, in the pathophysiology of BSP (Mascia et al., 2020). However, the precise pathophysiological mechanisms remain incompletely understood (Mascia et al., 2020; Hwang and Eftekhari, 2018).

Resting-state functional magnetic resonance imaging (rs-fMRI) is a promising non-invasive technique that measures brain function at rest *in vivo* by analyzing blood-oxygen-level-dependent (BOLD) signal fluctuations. This method has enabled researchers to study the pathophysiology of various neuropsychiatric disorders (Yang et al., 2020). Amplitude of low-frequency fluctuation (ALFF) is a reliable and sensitive rs-fMRI metric for quantifying the amplitude of spontaneous low-frequency oscillations (typically 0.01–0.1 Hz), reflecting the intensity of local neural activity in the brain during rest (Zang et al., 2007). To date, a few rs-fMRI studies have reported specific ALFF or fractional ALFF (fALFF) alterations associated with BSP. For instance, Yang et al. observed increased ALFF in the left orbitofrontal areas extending from the middle frontal gyrus to inferior frontal gyrus and decreased ALFF in the left thalamus in patients with BSP relative to healthy controls (HCs) (Yang et al., 2013). Another study by Zhou et al. showed that patients with BSP had increased ALFF in the left putamen, pallidum, insular lobe, and medial prefrontal cortex as well as decreased ALFF in the bilateral somatosensory regions, thalami, cerebellum, and medial and posterior cingulate cortex relative to HCs (Zhou et al., 2013). Feng et al. observed increased ALFF in the bilateral supplementary motor area, left cerebellar Crus I, and left fusiform gyrus, as well as decreased ALFF in the bilateral superior medial prefrontal cortex and right superior frontal gyrus (Feng et al., 2021). Patients with BSP also displayed increased static ALFF in the left primary motor cortex and increased dynamic ALFF variance in the right primary motor cortex compared to HCs (Luo et al., 2022). In contrast, increased fractional ALFF in the right caudate head was observed in patients with BSP (Ni et al., 2017). Furthermore, some of these (f) ALFF alterations were correlated with symptom severity and illness duration in BSP (Yang et al., 2013; Ni et al., 2017). These findings highlight the potential role of altered spontaneous brain activity in the pathophysiology of BSP. However, inconsistencies across these studies—potentially attributable to small sample sizes, demographic and clinical heterogeneity, medication effects, and methodological variations in image acquisition, preprocessing, and statistical analysis—preclude definitive conclusions. More evidence is thus required to delineate the patterns of spontaneous neural activity in BSP.

Furthermore, the most common treatment for BSP, botulinum toxin (BoNT) injections (Duarte et al., 2020), while primarily acting at the neuromuscular junction, has been shown to induce significant modulations in central nervous system activity and connectivity in related focal dystonias. Studies in cervical dystonia, for example, have demonstrated that BoNT therapy can alter resting-state cerebellar connectivity (Hok et al., 2021), change sensorimotor network activation (Nevrlý et al., 2018), and lead to partial normalization of widespread brain connectivity abnormalities, with some changes correlating with clinical improvement (Brodoehl et al., 2019; Feng et al., 2021). These central effects of BoNT suggest that investigations of treated patients may reflect a brain state already modified by therapeutic intervention. Therefore, studying medication-naïve patients with BSP is particularly important to characterize the intrinsic neural alterations underlying the disorder, uncontaminated by the central modulatory effects of BoNT. This approach allows for a clearer understanding of the fundamental pathophysiology of BSP.

Accordingly, the present study employed ALFF analysis of rs-fMRI data to investigate local neural activity abnormalities in medication-naïve patients with BSP compared to HCs. We hypothesized that altered brain activity in patients with BSP would be primarily located within the motor network and would correlate with both symptom severity and illness duration.

## Methods

### Subjects

Thirty-two patients with BSP diagnosed according to the established criteria (Defazio et al., 2013), and 32 age- and sex-matched HCs were recruited for this study. Only right-handed participants were included. Exclusion criteria for all participants included a past or present history of other neurological diseases, major psychiatric disorders, ophthalmologic diseases (beyond BSP for patients), alcohol or drug abuse, a family history of movement disorders, and evidence of structural lesions identified from conventional MRI examination. All patients were medication-naïve, having received no prior treatment for BSP at the time of enrollment or during MRI data acquisition. The Jankovic Rating Scale (JRS) was used to assess the severity (JRS severity subscale) and frequency (JRS frequency subscale) of eyelid spasms (Jankovic et al., 2009). At the time of image acquisition, all study participants underwent evaluation of depressive and anxiety symptoms using the self-rating depression scale (SDS) (Zung, 1965) and the self-rating anxiety scale (SAS) (Zung, 1971), respectively. In addition, sex, age, education level, and illness duration were recorded.

All participants provided written informed consent prior to inclusion. The study was approved by the Ethics Committee of the Affiliated Hospital 6 of Nantong University (Yancheng Third People’s Hospital) and conducted in accordance with the Declaration of Helsinki.

### MRI acquisition protocol

Brain rs-fMRI data were acquired using a 3.0-Tesla GE Discovery MR 750 scanner (Milwaukee, WI, USA) with an 8-channel phased-array head coil. A whole-brain gradient-recalled echo-planar imaging (GRE-EPI) sequence was used with the following scan parameters: repetition time/echo time = 2000 ms/30 ms, field of view = 240 mm × 240 mm, matrix = 64 × 64, flip angle = 90°, voxel size = 3.75 mm × 3.75 mm × 4.0 mm, number of slices = 35, slice thickness = 4 mm with no interslice gap, and time points = 230. Participants were instructed to lie still with their eyes closed, remain awake, and not think of anything in particular during the scan.

### Rs-fMRI data preprocessing

Rs-fMRI data preprocessing was performed using the Data Processing and Analysis of Brain Imaging (DPABI)^1^ toolbox (Yan et al., 2016) based on statistical parametric mapping software, version 12 (SPM12)^2^ runningunder MATLAB R2018b (The Mathworks, MA, USA). The process briefly included (a) data conversion from DICOM to NIFTI format; (b) removal of the first 10 volumes for signal equilibration; (c) slice timing for the remaining images to correct the differences in image acquisition time between slices; (d) realignment for head motion correction [with no more than 2.0 mm displacement or 2.0° in any direction and with average frame wise displacement (FD) <0.5 mm]; (e) spatial normalization to the Montreal Neurological Institute (MNI) EPI template (resampled voxel size = 3 × 3 × 3 mm^3^); (f) smoothed with 4 mm full-width at half-maximum Gaussian kernel; (g) nuisance covariates regression, including the global mean signal, white matter signal, cerebrospinal fluid signal and Friston 24 motion parameters; and (h) linear trend removal, and temporal filtering (0.01–0.1 Hz).

### ALFF analysis

ALFF analysis was performed using the DPABI toolbox (Yan et al., 2016). After preprocessing, the filtered time series for each voxel was transformed to the frequency domain using the Fast Fourier Transform. The power spectrum was then obtained, and the ALFF value for each voxel was calculated as the square root of the power spectrum averaged across the 0.01–0.1 Hz frequency band. For standardization, each voxel’s ALFF value was divided by the global mean ALFF value within a whole-brain mask, yielding a standardized whole-brain ALFF map for each participant.

### Statistical analysis

Demographic and clinical variables were compared between the BSP and HC groups using independent two-sample *t*-tests for continuous variables and chi-squared tests for categorical variables (sex). A *p* < 0.05 was considered statistically significant. These analyses were performed using SPSS 18.0 (IBM Corp., Armonk, NY, USA).

The statistical analysis of voxel-based ALFF differences between groups was performed using DPABI (Yan et al., 2016). An independent two-sample *t*-test was performed, controlling for age, sex, education level, SAS scores, SDS scores, and FD. Statistical significance was determined using threshold-free cluster enhancement (TFCE) with family-wise error (FWE) correction for multiple comparisons (*p* < 0.05). An additional cluster extent threshold of >10 contiguous voxels was applied to significant clusters (Chen et al., 2018).

Partial correlation coefficients between the ALFF values extracted from the significantly altered brain regions and the clinical variables (illness duration, JRS-total score, JRS-severity subscale, and JRS-frequency subscale) in patients with primary blepharospasm were further calculated, controlling for age, sex, education level, SAS scores, SDS scores, and FD. The significance level was initially set at *p* < 0.05. Multiple comparisons were further adjusted using Bonferroni corrections, with the significance threshold set at *p* < 0.05/[4 × 5], where 4 represents the number of clinical variables and 5 represents the number of altered ALFF regions.

## Results

### Demographic and clinical characteristics

Table 1 summarizes demographic and clinical characteristics of the participants. No significant differences were found between medication-naïve BSP patients and HCs in age (*p* = 0.08), gender (*p* = 0.8), education level (*p* = 0.92), or mean FD (*p* = 0.42). However, SDS and SAS scores were significantly higher in the BSP group compared to HCs (*p* = 0.001 and *p* = 0.001, respectively).

**Table 1:** Demographic and clinical characteristics of all subjects.

Characteristics	Primary blepharospasm (*n* = 32)	Healthy controls (*n* = 32)	*p*
Age (year)	52.53 (5.9)	50.06 (5.3)	0.08^a^
Gender (male/female)	13/19	14/18	0.8^b^
Education (year)	9.03 (3.65)	9.13 (4)	0.92^a^
Illness duration (month)	12.81 (5.34)	–	–
JRS-total	3 (2, 4)^c^	–	–
JRS-severity subscale	1 (1, 2)^c^	–	–
JRS-frequency subscale	2 (1, 2)^c^	–	–
SAS	42.15 (7.41)	36.62 (5.22)	0.001^a^
SDS	43.63 (6.83)	38.09 (5.43)	0.001^a^
Mean FD (mm)	0.13 (0.06)	0.12 (0.06)	0.42^a^

JRS, Jankovic Rating Scale; SAS, Self-rating anxiety scale; SDS, Self-rating depression scale; FD, framewise displacement. ^a^The *p*-values were obtained by two-sample *t*-tests. ^b^The *p-*value for gender distribution was obtained by chi-square test. ^c^Data was presented as median (interquartile range). Data was presented as mean (standard deviation) unless otherwise specified.

### Group differences in ALFF

**Figure 1:**
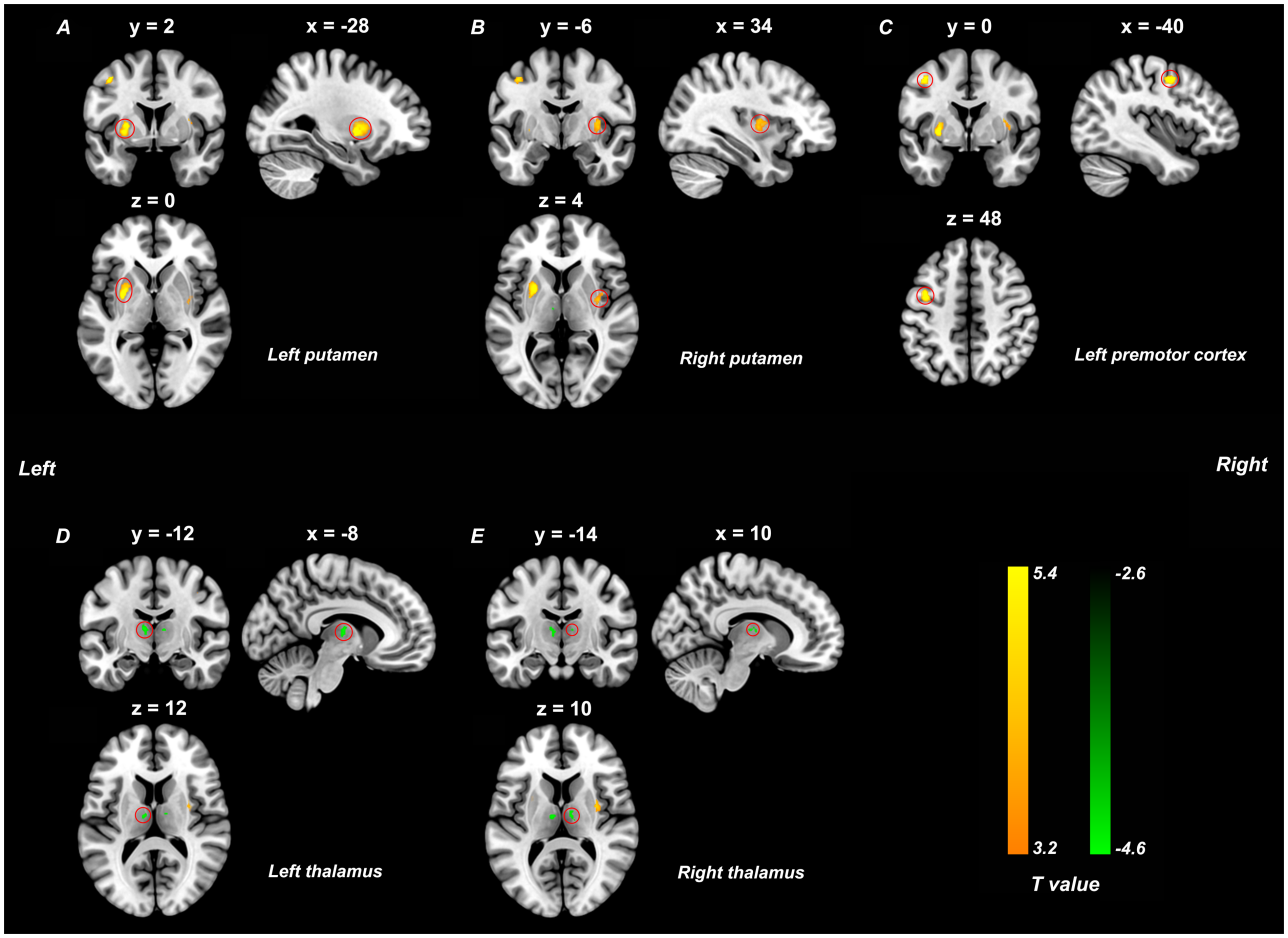
ALFF differences between the primary blepharospasm and the healthy control groups (*p* < 0.05, TFCE FWE corrected, cluster size > 10 voxels). Yellow brain regions **(A–C)** represent increased ALFF values and green regions **(D,E)** indicate decreased ALFF in the primary blepharospasm group compared to the healthy control group. The color bar represents the *t*-values of the group analysis. ALFF, amplitude of low-frequency fluctuation; TFCE, threshold free cluster enhancement; FWE, family-wise error.

Compared to HCs, the BSP group exhibited significantly increased ALFF in the bilateral putamen and left premotor cortex, and decreased ALFF in the bilateral thalamus (Table 2 and Figure 1). These results were obtained after controlling for age, sex, education level, mean FD, SDS scores, and SAS scores (*p* < 0.05, TFCE FWE corrected).

**Table 2:** Clusters with significant ALFF differences between patients with primary blepharospasm and healthy controls.

Brain region (BA)	MNI coordinate			Size (voxels)	Peak *t-*value
	x	y	z		
Primary blepharospasm > healthy controls					
Left putamen	−28	2	0	184	5.4
Right putamen	34	−6	4	121	4.2
Left premotor cortex (BA6)	−40	0	48	96	5.3
Primary blepharospasm < healthy controls					
Left thalamus	−8	−12	12	22	−4.6
Right thalamus	10	−14	10	14	−4.1

ALFF, amplitude of low-frequency fluctuation; BA, Brodmann area; MNI, Montreal Neurological Institute.

### Correlation analysis

**Figure 2:**
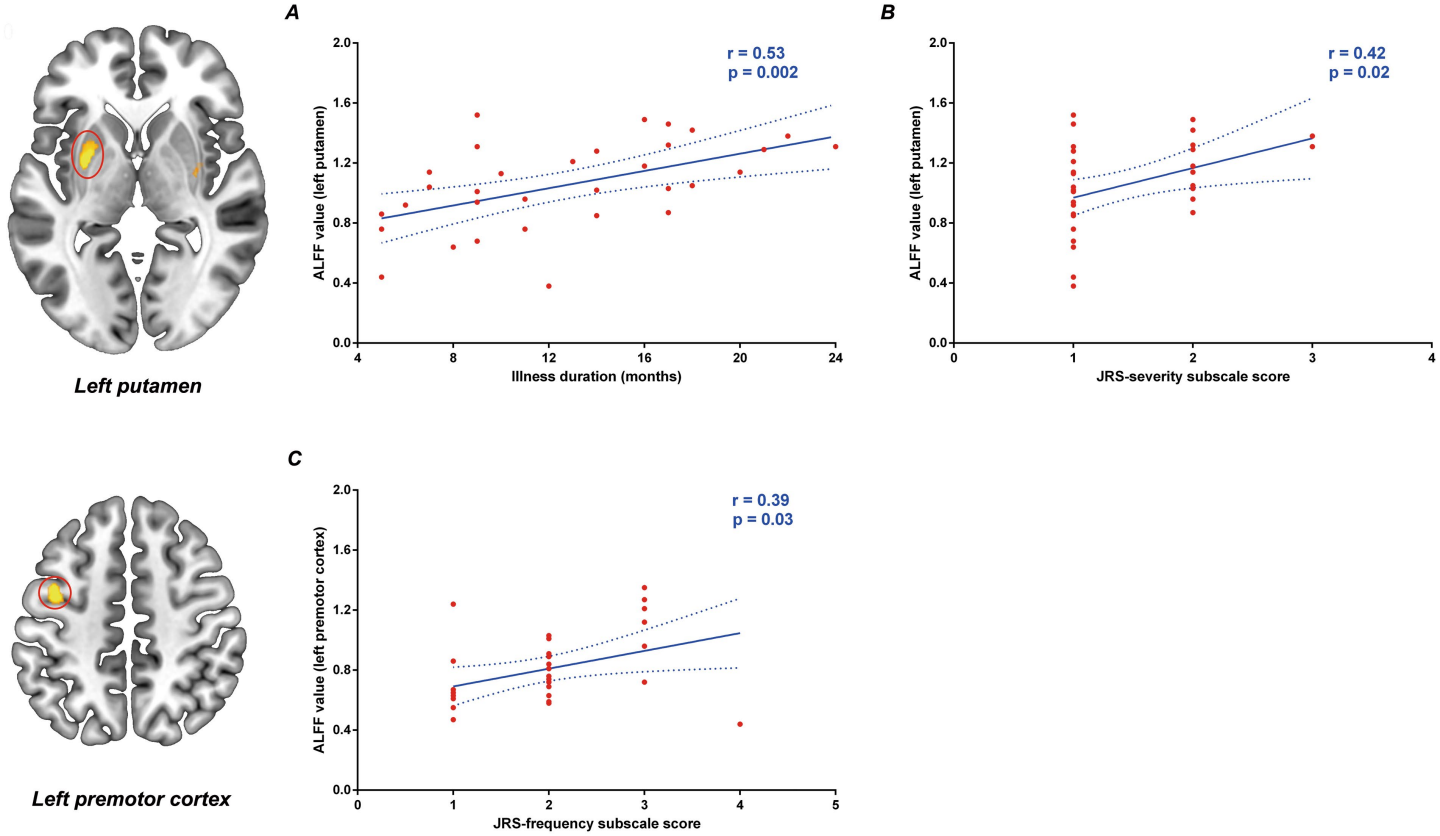
Correlation between the abnormal ALFF values and clinical variables in primary blepharospasm patients. ALFF values in the left putamen were positively correlated with the illness duration **(A)** and the JRS severity subscale score **(B)**. ALFF values in the premotor cortex was positively correlated with the JRS frequency subscale score **(C)**. ALFF, amplitude of low-frequency fluctuation; JRS, Jankovic rating scale.

The ALFF values in the left putamen in the patient group were positively correlated with illness duration (*r* = 0.53, *p* = 0.002, Figure 2A) and JRS severity subscale score (*r* = 0.42, *p* = 0.02, Figure 2B) controlling for age, gender, education level, mean FD, SDS score and SAS score. Our study also showed a positive correlation between ALFF values in the premotor cortex and JRS frequency subscale score (*r* = 0.39, *p* = 0.03, Figure 2C). However, after applying multiple comparison corrections (adjusted *p* < 0.0025, Bonferroni correction), only the positive correlation between left putamen ALFF and illness duration remained statistically significant.

## Discussion

The present study utilized ALFF analysis of rs-fMRI data to investigate spontaneous neural activity in medication-naïve BSP patients. We found increased ALFF in the bilateral putamen and left premotor cortex, and decreased ALFF in the bilateral thalamus in BSP patients compared to HCs, indicating abnormal intrinsic activity in these key motor-related regions. Furthermore, our study demonstrated a significant positive correlation between ALFF values in the left putamen and illness duration in BSP patients.

BSP, characterized by involuntary eyelid spasms, has traditionally been viewed as a basal ganglia disorder (Defazio et al., 2017; Mascia et al., 2020). Structural lesions in the basal ganglia have indeed been associated with secondary blepharospasm (Defazio et al., 2017). Our rs-fMRI findings of abnormal spontaneous activity in the bilateral putamen, thalamus, and left premotor cortex provide compelling in-vivo support for the involvement of the cortico-striato-thalamo-cortical (CSTC) circuits, which is critical for motor planning, control, and execution (Fujita and Eidelberg, 2017; Simonyan et al., 2017; Huang et al., 2017; Jinnah et al., 2017). Previous literature has proposed that focal dystonia, including BSP, may be driven by imbalances in excitation and inhibition within these motor circuits. Specifically, increased excitation in the direct pathway and decreased inhibition in the indirect pathway could result in hyperexcitability within these circuits (Fujita and Eidelberg, 2017; Simonyan et al., 2017; Huang et al., 2017; Jinnah et al., 2017). However, the pattern of decreased thalamic ALFF alongside increased premotor ALFF seemed paradoxical under older classic linear models (Vitek, 2002) that thalamic hypoactivity should lead to reduced cortical activity. These findings may best interpreted through the lens of the contemporary “network model” of dystonia, which conceptualizes the disorder as a multi-faceted circuit failure (Mascia et al., 2020; Jinnah et al., 2017; Neychev et al., 2011). The decreased thalamic ALFF likely reflects not simple hypoactivity, but a pathological shift to a low-frequency, burst-firing pattern, which degrades the quality of information sent to the cortex (Vitek, 2002; Zhuang et al., 2004). Concurrently, the increased premotor ALFF is driven by a “perfect storm” of cortical hyperexcitability due to intrinsic loss of inhibition (Balint et al., 2018) and a hyperfunctional basal ganglia “direct pathway” that provides a powerful, independent excitatory drive to the cortex (Simonyan et al., 2017). Therefore, our fMRI findings provide a compelling *in-vivo* snapshot of this multi-faceted network failure. This interpretation is further corroborated by a wealth of evidence from brain lesion studies, neurophysiological investigations, and other imaging modalities that consistently implicate these same nodes (Defazio et al., 2017; Mascia et al., 2020; Jinnah et al., 2017). Furthermore, some of these brain areas, such as the putamen, thalamus, and premotor cortex are implicated as therapeutic targets for treating other forms of focal dystonia (Jinnah et al., 2017). While these alterations provide valuable insight, further studies incorporating differential diagnostic paradigms and validation of classification metrics (e.g., accuracy, robustness) are warranted to establish their utility as reliable diagnostic or prognostic biomarkers for BSP. The specific pattern of ALFF changes in the putamen, thalamus, and premotor cortex—key elements of the sensorimotor network—highlights the complex multi-regional nature of BSP pathophysiology, reframes our understanding beyond simple linear models, and offers new avenues for targeted intervention and future research.

The findings from our study are not uniformly consistent with all prior ALFF/fALFF investigations in BSP, a situation that is common in a developing field of research. A systematic review of these studies, summarized in Supplementary Table 1, helps to contextualize these apparent discrepancies by highlighting significant heterogeneity in patient cohorts, treatment status, and methodology (Yang et al., 2013; Zhou et al., 2013; Feng et al., 2021; Luo et al., 2022; Ni et al., 2017). One explanation for these discrepancies is the understanding of BSP as a network disorder; dysfunction in any node or their interactions in this complex network may result in the expression of dystonic movement. Additionally, the notable clinical and etiological heterogeneity of BSP, including its varied motor and non-motor manifestations, likely contributes to heterogeneous neural substrates (Defazio et al., 2017). As previously mentioned, methodological differences (e.g., image acquisition, preprocessing steps like global signal regression, statistical thresholds), sample size limitations, and potential therapeutic confounds in other studies may also contribute to these inconsistencies. The present study mitigated some of these factors by including only medication-naïve patients, controlling for depression and anxiety scores (which differed between groups), and recruiting a relatively larger cohort characterized by younger mean age, shorter illness duration, and milder illness severity compared to some previous reports. These factors collectively contribute to the variability in reported findings and underscore the importance of our carefully controlled study design.

Despite some inconsistencies, a consistent decrease in thalamic ALFF was identified in our study and two other ALFF studies (Yang et al., 2013; Zhou et al., 2013), and increased putaminal ALFF was observed in our study and another ALFF study (Zhou et al., 2013) in in BSP patients. Our study showed that bilateral thalamic ALFF values did not correlate with illness severity or duration, indicating that this alteration might represent an early or core feature of the disease. These consistent findings suggest that decreased ALFF in the thalamus may be a relatively robust imaging feature for BSP. Conversely, the positive correlation between left putamen ALFF and illness duration (and a trend with JRS severity before Bonferroni correction) suggests this alteration might reflect disease progression, maladaptive plasticity, or compensatory mechanisms over time. Our novel finding of increased ALFF in the left premotor cortex, which was not a consistent feature in previous reports, may be particularly insightful. Its emergence in our specific cohort of untreated, relatively early-stage patients could highlight an initial cortical response to the underlying circuit imbalance, a feature that might be obscured by chronicity or treatment effects in other cohorts. The trend toward a correlation with spasm frequency further suggests that this premotor hyperactivity could be directly linked to the expression of motor symptoms, emphasizing its role in BSP pathophysiology.

Interestingly, a noteworthy and important finding of our study is the unilateral nature of some of our key results—specifically, the increased ALFF in the left premotor cortex and the correlation between illness duration and ALFF in the left putamen—despite the clinically bilateral presentation of BSP. This apparent discrepancy warrants careful consideration and is best interpreted in the context of both hemispheric dominance for motor control and the existing literature. Our cohort was predominantly right-handed, meaning the left cerebral hemisphere is dominant for planning and executing motor tasks. Crucially, our unilateral finding is not an anomaly in the BSP literature; rather, it contributes to an emerging pattern of asymmetric findings. As detailed in Supplementary Table 1, prior studies have frequently reported lateralized hyperactivity. For instance, Zhou et al. also found predominantly left-lateralized increases in the putamen and pallidum (Zhou et al., 2013), while Luo et al. reported increased ALFF specifically in the left primary motor cortex (Luo et al., 2022). The recurrence of left-hemisphere motor circuit hyperactivity across multiple independent studies lends substantial weight to the hypothesis that the dominant hemisphere is uniquely affected or more reactive in dystonia. This same rationale extends to the left-lateralized correlation with illness duration. The putamen is a critical site for procedural learning; it is physiologically plausible that progressive, long-term maladaptive plasticity would be most evident in the most heavily utilized cortico-striatal circuit of the dominant hemisphere. Furthermore, we cannot exclude the possibility of a statistical thresholding effect, where a similar but weaker effect in the right hemisphere may not have survived our stringent correction. Nevertheless, the consistency of left-sided motor circuit findings across several studies, including our own, strongly suggests that hemispheric dominance plays a key role in the functional neuroanatomy of BSP. Future studies that systematically record handedness and symptom laterality are essential to further elucidate this important aspect of the disease.

### Limitations

Several limitations should be noted in the present study. Firstly, like most other cross-sectional neuroimaging studies, our study cannot elucidate the causal relationship between ALFF alterations and BSP symptoms or determine if these changes are primary or secondary to the disease process. Longitudinal studies are needed to address this. Secondly, while BSP exhibits a female predominance and we controlled for sex as a covariate in the analysis, further studies with larger, sex-stratified samples are needed to explore potential sex-specific brain functional alterations in BSP. Thirdly, the ALFF is widely considered to be a marker of spontaneous neuronal activity, but its direct physiological basis remains an area of active investigation. The ALFF metric itself only quantifies the strength of spontaneous regional neural activity, and does not directly capture excitatory/inhibitory dynamics, nor the directionality or functional connectivity between regions. Therefore, while our network-based interpretation is plausible, we cannot conclusively determine the pathophysiological mechanisms or the flow of information within the implicated networks. Future studies combining ALFF with other rs-fMRI metrics, such as functional connectivity, effective connectivity (e.g., Granger causality analysis, dynamic causal modeling), and measures of regional homogeneity, could provide deeper insights into the roles of these brain regions and their interactions in BSP. Fourthly, ALFF is susceptible to physiological noise, and even subtle head movements can artificially inflate ALFF signals, particularly in subcortical regions such as the putamen and thalamus. While we applied strict motion correction, excluded participants with excessive motion, and included FD as a covariate in our analyses, it is not possible to fully eliminate residual motion effects. Moreover, we did not perform direct visualization or video assessment of facial spasms during scanning, which might have helped to further control for symptom-related motion and thus represents another methodological limitation. Finally, our study employed a single imaging modality (resting-state fMRI) and one analytic approach (ALFF). The addition of multi-modal imaging approaches (e.g., structural MRI, diffusion tensor imaging, or PET), task-based paradigms, and integration of other data (such as genetics or electrophysiology), alongside simultaneous electromyography-fMRI acquisition, would help further elucidate the complex neuropathophysiology of BSP by clarifying the relationship between peripheral muscle contractions and central neural patterns, thereby disentangling primary disease processes from secondary compensatory or adaptive mechanisms.

## Conclusion

In conclusion, our study identified significant ALFF alterations in the bilateral putamen, left premotor cortex, and bilateral thalamus in medication-naïve BSP patients compared to HCs. The correlation of left putamen ALFF with illness duration suggests a dynamic role for this region in the disease course. These findings highlight dysfunction within key nodes of the motor network and contribute to a deeper understanding of the neuropathophysiology of BSP, particularly in its untreated state.

## Data Availability

The original contributions presented in the study are included in the article/Supplementary material, further inquiries can be directed to the corresponding author.

## Footnotes

^1^ http://rfmri.org/DPABI

^2^ http://www.fil.ion.ucl.ac.uk/spm/software/spm12
