# Supplementary material for: Spontaneous neural activity alterations in medication-naïve primary blepharospasm: a resting-state functional magnetic resonance imaging study
Source: Front Syst Neurosci. 2025 Jul 14;19:1639915. doi: 10.3389/fnsys.2025.1639915 (PMC12301356; doi:10.3389/fnsys.2025.1639915)
Supplement: Supplementary file 1 [file Table_1.docx]

**Supplementary Table 1. ALFF/fALFF studies in primary blepharospasm**

|  | Zhou et al., 2013 (1) | Yang et al., 2013 (2) | Ni et al., 2017 (3) | Feng et al., 2021 (4) | Luo et al., 2022 (5) | The present study |
| --- | --- | --- | --- | --- | --- | --- |
| Samples of patients/HCs | 9 / 9 | 18 / 18 | 26 / 26 | 25 / 23 | 46 / 46 | 32 / 32 |
| Age of patients | 61.7 (52–66) years | 55.54 (8.42) years | 56.5 (12.2) years | 49.68 (8.41) years | 53 (34-69) years | 52.5 (5.9) years |
| Illness duration | 2.7 (1.8) years | 3.83 (3.93) years | 50.23 (21.78) months | 11.64 (9.01) months | 5.5 (0.5–23) years | 12.81 (5.34) months |
| JRS | Not available | JRS-total: 5.22 (1.44)  JRS-severity: 2.67 (0.69)  JRS-frequency: 2.56 (0.86) | JRS-total: 6 (3)  JRS-severity: 3 (2)  JRS-frequency: 3 (1) | JRS-severity: 2.64 (0.81) | JRS-total: 6 (2–8)  JRS-severity: 3 (1–4)  JRS-frequency: 3 (1–4) | JRS-total: 3 (2–4)  JRS-severity: 1 (1–2)  JRS-frequency: 2 (1–2) |
| Anxiety/ depressive symptoms | Not available | Not available | Not available | SAS: 42.52 (10.18)  SDS: 48.51 (8.86) | Not available | SAS: 42.15 (7.41)  SDS: 43.63 (6.83) |
| Scanner | 3.0T GE-Excite | 3.0T GE-Excite | 3.0T GE-Signa | 3.0T Siemens | 3.0T Siemens | 3.0T GE Discovery 750 |
| Image properties | TR/TE: 2000/30ms; Slices: 30; Thickness: 5 mm; Voxel: 3.75× 3.75× 5 mm³ | TR/TE: 2000/30ms;Slices: 30; Thickness: 5 mm; Voxel: 3.75× 3.75× 5 mm³ | TR/TE: 3000/30ms; Slices: 32; Thickness: 4 mm | TR/TE: 2000/30ms; Slices: 30; Thickness: 4 mm | TR/TE: 2000/30ms; Slices:33; Voxel: 3.44 × 3.44 × 3 mm³ | TR/TE: 2000/30ms; Slices: 35; Thickness: 4mm; Voxel: 3.75×3.75×4 mm³ |
| Analytic methods | ALFF | ALFF | fALFF | ALFF | ALFF | ALFF |
| Statistical analysis | p < 0.001, uncorrected | p < 0.05, FWE corrected | p < 0.05, AlphaSim corrected | p < 0.05, GRF corrected | p < 0.05, GRF corrected | p < 0.05, TFCE-FWE corrected |
| Imaging results | Increased: L-putamen, L-insula, L-pallidum, bilateral mPFC; Decreased:  bilateral thalamus, bilateral cerebellum, bilateral post/precentral gyrus, PCC | Increased: bilateral orbitofrontal areas;  Decreased: bilateral thalamus | Increased: R-caudate head | Increased: bilateral SMA, L-cerebellum, L-fusiform; Decreased: bilateral superior MPFC, R-SFG, R-IFG | Increased: left primary motor cortex, right inferior parietal gyrus, and bilateral MFG; Decreased: right precuneus, left postcentral gyrus | Increased: bilateral putamen, L-premotor cortex; Decreased: bilateral thalamus |
| Treatment | Two patients received BoNT injections 6 months before the study, but symptoms recurred after 3 months | Not received receiving medications for about 24h prior to MRI scanning | No history of treatment with psychotropic drugs or neuroleptic drugs, and no treatment with BoNT for at least 3 months prior to MRI scanning | Not received BoNT or any medication for dystonia or mental illness within 3 months before enrollment | BoNT treatment for at least 3 months post-injection | Strictly medication-naïve |

ALFF, amplitude of low-frequency fluctuation; fALFF, fractional ALFF; HCs, healthy controls; JRS, Jankovic Rating Scale; TR, repetition time; TE, echo time; FWE, family wise error; GRF, Gaussian random field theory; TFCE, threshold free cluster enhancement; SDS, self-rating depression scale; SAS, self-rating anxiety scale; BoNT, botulinum neurotoxin; L, left; R, right; mPFC, medial prefrontal cortex; PCC, posterior cingulate cortex; SMA, supplementary motor area; SFG, superior frontal gyrus; IFG, inferior frontal gyrus

**References**

1. Zhou B, Wang J, Huang Y, Yang Y, Gong Q, Zhou D. A Resting State Functional Magnetic Resonance Imaging Study of Patients With Benign Essential Blepharospasm. Journal of Neuro-Ophthalmology. 2013;33(3):235-40.

2. Yang J, Luo C, Song W, Chen Q, Chen K, Chen X, et al. Altered regional spontaneous neuronal activity in blepharospasm: a resting state fMRI study. Journal of Neurology. 2013;260(11):2754-60.

3. Ni M-F, Huang X-F, Miao Y-W, Liang Z-H. Resting state fMRI observations of baseline brain functional activities and connectivities in primary blepharospasm. Neuroscience Letters. 2017;660:22-8.

4. Feng C, Jiang W, Xiao Y, Liu Y, Pang L, Liang M, et al. Comparing Brain Functional Activities in Patients With Blepharospasm and Dry Eye Disease Measured With Resting-State fMRI. Frontiers in Neurology. 2021;12.

5. Luo Y, Guo Y, Zhong L, Liu Y, Dang C, Wang Y, et al. Abnormal dynamic brain activity and functional connectivity of primary motor cortex in blepharospasm. European Journal of Neurology. 2022;29(4):1035-43.
